# Supplementary material for: Accuracy of short tandem repeats genotyping tools in whole exome sequencing data
Source: F1000Res. 2020 Mar 23;9:200. [Version 1] doi: 10.12688/f1000research.22639.1 (PMC7327730; doi:10.12688/f1000research.22639.1)
Supplement: Peer review history

# Peer review: Reviewer response for version 1

**DOI:** 10.5256/f1000research.24995.r63748

**Published:** 2020-06-30

**Authors:** Maria Anisimova

**Affiliations:** 1 Institute of Applied Simulation, School of Life Sciences and Facility Management, Zurich University of Applied Sciences (ZHAW), Waedenswil, Switzerland; 2 Swiss Institute of Bioinformatics, Lausanne, Switzerland

In recent years short tandem repeats (STRs) drew increased attention because of their frequent associations with disease, as well as their abundance and a diversity of roles in protein functions. Consequently, many methods were developed for detecting and genotyping tandem repeats. With wide variation in methodology, and its related settings, it is often hard to know which software can provide an optimal performance and be integrated in bioinformatics pipelines. The study by Halman and Oshlack “Accuracy of short tandem repeats genotyping tools in whole exome sequencing data” contributes some insights for scientists who wish to study short tandem repeats (STRs) loci in genomic data. The article is clearly written and should be practically useful for those developing relevant bioinformatics pipelines.

 The authors evaluate four STR genotyping tools, they provide a clear description and reasoning for their choices and point out the main differences between the tools. Overall, the results are not surprising and could be expected:  the evaluated tools all have different strengths and weaknesses and the choice should be driven by the specific goals of the analysis.  Clearly, different filtering settings will produce results of different accuracy, with a trade-off between the genotyping accuracy and the highest number of calls. As expected, homorepeats and dinucleotide repeats are the most problematic and there is currently no tool providing a good solution for such STRs. Apart from the valuable main message, the quantitative results should not be generalized, these are more to get a “feeling” of the expected accuracy than any guarantee. Indeed, it is difficult to directly compare methods that differ in so many ways: conceptually, by assumptions of random noise model, alignment procedures, filtering options and cut-offs, etc. Nevertheless, I applaud the authors for trying to accomplish (at least partially) this difficult task.

 At the same time, I would like to raise some points for reflection.

 Note, that all evaluated tools required a set of defined STRs loci. These were detected using the TRF tool (Benson 1999) ^1^ based on self-alignment. In our article Schaper *et al.* (2012) ^2^, we have evaluated several tandem repeat (TR) predictors, including TRF. As one can expect (and similar to the findings in the article reviewed above), due to the heterogeneity of the methods in terms of their main concepts and assumptions, we observed a wide spectrum of variable performances over the space of TRs. Specifically, for TRF, we observed that this predictor is very conservative – having a low false positive rate across the TR space (in terms of TR unit numbers and unit length) but also a very low power (high false negative rate). For repeats with very short units, TRF is extremely conservative. Therefore, such bias in STR detection by TRF may have consequences on the observed by the authors distribution of STR unit sizes (e.g., in Figure 1b, the overrepresentation of longer 6-nt units may be explained simply by the TRF-specific bias).

 Therefore, I would be curious how the results of Halman and Oshlack would change, both relatively and quantitatively, were they to use a TR annotation tool with contrasting properties, i.e. that is better at detecting STRs specifically. For example, this could be Xtreme (Newman and Cooper, 2007) ^3^ or a meta TR predictor, each followed by statistical filtering (as we suggest in Schaper *et al *2012 ^2^ and also in Anisimova *et al. *2015) ^4^.  These approaches typically suggest a TR unit distribution dominated by homo- and dinucleotide repeats (eg, see Delucchi *et al*. 2020 ^5^).

 Finally, the authors also mention the current gap in methodology for indel calling. For repeat regions indels become particularly important - as a consequence of TR unit number variation, but also due to regular indel events. In this respect, while methods to tackle this problem are not available, one should be aware of this potential problem for their analyses, interpretation of results and overall conclusions.

Is the work clearly and accurately presented and does it cite the current literature?

Yes

If applicable, is the statistical analysis and its interpretation appropriate?

Yes

Are all the source data underlying the results available to ensure full reproducibility?

Yes

Is the study design appropriate and is the work technically sound?

Yes

Are the conclusions drawn adequately supported by the results?

Yes

Are sufficient details of methods and analysis provided to allow replication by others?

Yes

Reviewer Expertise:

computational molecular evolution, bioinformatics

I confirm that I have read this submission and believe that I have an appropriate level of expertise to confirm that it is of an acceptable scientific standard.

## References

[1] : Tandem repeats finder: a program to analyze DNA sequences. *Nucleic Acids Res*.1999;27(2) : 10.1093/nar/27.2.573 573-80 10.1093/nar/27.2.573 9862982PMC148217

[2] : Repeat or not repeat?--Statistical validation of tandem repeat prediction in genomic sequences. *Nucleic Acids Res*.2012;40(20) : 10.1093/nar/gks726 10005-17 10.1093/nar/gks726 22923522PMC3488214

[3] : XSTREAM: a practical algorithm for identification and architecture modeling of tandem repeats in protein sequences. *BMC Bioinformatics*.2007;8: 10.1186/1471-2105-8-382 382 10.1186/1471-2105-8-382 17931424PMC2233649

[4] : Statistical approaches to detecting and analyzing tandem repeats in genomic sequences. *Front Bioeng Biotechnol*.2015;3: 10.3389/fbioe.2015.00031 31 10.3389/fbioe.2015.00031 25853125PMC4362331

[5] : A New Census of Protein Tandem Repeats and Their Relationship with Intrinsic Disorder. *Genes (Basel)*.2020;11(4) : 10.3390/genes11040407 10.3390/genes11040407 32283633PMC7230257

# Peer review: Reviewer response for version 1

**DOI:** 10.5256/f1000research.24995.r63745

**Published:** 2020-06-17

**Authors:** Jan Radvanszky, Jaroslav Budiš

**Affiliations:** 1 Institute of Clinical and Translational Research, Biomedical Research Center, Slovak Academy of Sciences, Bratislava, Slovakia; 2 Science Park, Comenius University in Bratislava, Bratislava, Slovakia; 3 Geneton Ltd., Bratislava, Slovakia; 4 Slovak Centre of Scientific and Technical Information, Bratislava, Slovakia

Andreas Halman and Alicia Oshlack prepared and submitted a very interesting manuscript dealing with the results of their comparison of four selected STR genotyping tools (LobSTR, HipSTR, GangSTR and RepeatSeq), which are designed to process data from massively parallel sequencing (here used for exome sequencing data sets). More precisely, they compared five tools in the initial phases of the experiments, however, GATK HaplotypeCaller is not a dedicated STR tool and was disregarded in later phases of the study because of its several limitations. Although the study is well designed, well evaluated and also well written, it has one substantial limitation - the study design itself. The authors chose an elegant and low-cost, but still high-throughput approach that relies on the biological fact that healthy males (at least in humans and other mammals) bear one X chromosome in their genome, so they are hemyzygous for the major part of this chromosome (when not considering the pseudoautosomal regions). Based on this phenomenon, they “ *expect(*ed) *all calls *(coming from the X chromosome) *to be homozygous*” therefore they evaluated the proportion of “ *incorrect calls revealed by a heterozygous call*” to determine the “ *accuracy of genotyping*”. Genotyping accuracy, however, has several aspects and the number of incorrectly called heterozygous positions (i.e. the false heterozygous rate) is only one part of it. The approach chosen by the authors, therefore, does not allow to determine the total genotyping accuracy, only the rate of false heterozygous calls, i.e. the ability of tools to discriminate between heterozygous and homozygous state. We need to mention here, that this limitation is clear from the study and is properly acknowledged by the authors in several places in the manuscript (both in the Introduction and also in the Discussion). We would like to emphasize, therefore, that we do not consider this limitation for an insufficiency of the study design. On the other hand, because of the inadequate wording chosen by the authors ( *genotyping accuracy*) we consider the conclusions described by these words only partly supported. 

 We agree with the authors, however, that they present a “ *different but complementary approach*” to evaluate the performance of STR genotyping tools, at least with respect to their ability to make a call, and to correctly assign homozygous calls to homozygous STR positions. We are sure that this approach will become a well established approach not only in future research studies, but also in everyday practice. Following thorough review of the manuscript, we would like to suggest some major and several minor points to be considered by the authors:

           

 Major suggestions:

1. We would like to suggest rewording of “ *genotyping accuracy*” (and similar formulations, such as “ *accuracy of genotyping*”, “ *performance of genotyping tools*”, etc.) with a more appropriate formulation - throughout the manuscript, everywhere, where they are used with respect to the results of the authors evaluation. Maybe with “ *false heterozygous rate*”? 
2. Page 10; Part “ *Effects of repeat unit and quality scores on accuracy*” - the conclusions, according to which: 
  - In case of GangSTR “ *We can also see that the accuracy of 1 bp repeat calls improves with the highest quality score (1.0) and a stronger filter for this repeat unit may be appropriate”*, seems not to be correct, since there is a relatively wide range of lower quality scores in which GangSTR leads to lower false heterozygous rates (and also with larger dot sizes) - at least according to Figure 6a
  - For HipSTR and 1 bp repeats there is a similar exception from the general trend that “ *keeping only the ones with the highest quality score will improve overall accuracy*”  
3. Figure 5 and results for the dinucleotide repeats: 
  - Why is the GC/CG dinucleotide missing from the compared possible dinucleotide sequences? If it is by mistake, it would be useful to add this dinucleotide to the comparison. If it has a specific reason, this should be discussed in the text.
  - Similarly to dinucleotides, there were also higher error rates found for homopolymers. It would be interesting to try to evaluate the error rate also for each of the four possible homopolymers and to see whether there are different trends between them (like the marked AC/TG trend with GangSTR and LobSTR). 

Minor suggestions:

1. Page 3; First indent of Introduction: with regard to the standard methods to genotype STRs it would be better to change “gel electrophoresis” to “capillary electrophoresis”, since gel electrophoresis most commonly refers to agarose or polyacrylamide gels
2. Page 6; Third and fourth indents of the part called “ *Dataset*”: We would suggest to merge the two parts dealing with GATK (“ *GATK-HC reported a median...*” and “ *GATK only makes calls at positions...*”) into one, because they are now unnecessarily separated by the part dealing with the “ *...23 loci for which the reference STR length was longer…*”  
3. Figure 2.: We recommend to: 
  - Use the same order of tools in the part A) and B) of the figure 
  - Include GATK in the charts, since GATK was also evaluated and compared in this step of the study (even if it resulted in small numbers of calls and its numbers does not necessarily reflect its ability to call genotypes, since it calls only alleles which are different from the reference genome). Although this will most probably lead to “simple rows” in the lower parts of the chart, because of the significantly lower numbers obtained with GATK, we believe that such inclusion will strengthen the visual recognition of the significant differences between the tools 
  - We believe also that adding the respective numerical values for each tool (for example up to the top of each bar in the bar chart, and under the median in the box-plot) can further enhance the informational value of the charts 
  - Provide also comparison of number of calls after applying the optimal filters (mentioned at the end of the “ *Dataset*” section) 
4. Page 7; Part “ *Effects of repeat unit and coverage on accuracy*”: 
  - With few exceptions, nearly each experiment of the study is visualized through plots. We think that the number of heterozygous calls as a function of repeat unit length for each compared tool would deserve a plot too, since we believe that readers would appreciate the visualisation of these results  
  - We think that the last sentence on the page (“ *While dinucleotide repeats showed a trend towards a lower error rate with increasing coverage for RepeatSeq and LobSTR, the trend was reversed for GangSTR*”) does not describe accurately the complex trends visualised by the plots on Fig.4 (for LobSTR and HipSTR). LobSTR for example shows a lowering trend only up to a certain point, where it sharply increases. This seems to be, moreover, an opposite trend to that seen for HipSTR, which has a rather increasing error rate (although with little fluctuations) up to a certain point when it suddenly drops 
  - Do the authors have an explanation for the sudden change in trends encountered in the LobSTR and HipSTR results for mono- and dinucleotide repeats? 
5. Figure 4. (and other line plots): The lines in the plots are difficult to read, especially in a printed form. We suggest reworking of the graphs for better clarity, for example with thicker trend lines, white background instead of grey, etc.
6. Figure 6: Can the authors include an explanation (or at least a hypothetical reason) to the “strange” trend encountered in the HipSTR results? 
7. Page 13; The very last sentence of the “ *Results*” section: We recommend to specify the respective time also for RepeatSeq, determined after the utilisation of more cores ( *while RepeatSeq´s time improved to ....*) 
8. Although they comprise only a relatively small part of X chromosome, the pseudoautosomal regions (PAR1 and PAR2) should be mentioned in the study. Together with the information, whether these regions were excluded or included in the target regions for the present study - alternatively, whether any of the called STR loci mapped to these regions (and if yes, then their proportion to the remaining region should be mentioned) 
9. Page 13; “ *Discussion*”: We suggest to move the actual fifth indent (“ *Here, we have presented one way of performing...*”) after the first one (“ *We used a novel way...*”), because together they will form a more consistent “introduction” to Discussion.

Is the work clearly and accurately presented and does it cite the current literature?

Yes

If applicable, is the statistical analysis and its interpretation appropriate?

Yes

Are all the source data underlying the results available to ensure full reproducibility?

Yes

Is the study design appropriate and is the work technically sound?

Yes

Are the conclusions drawn adequately supported by the results?

Partly

Are sufficient details of methods and analysis provided to allow replication by others?

Yes

Reviewer Expertise:

JR - human molecular genetics, genomic technologies; JB - bioinformatics, computational biology

We confirm that we have read this submission and believe that we have an appropriate level of expertise to confirm that it is of an acceptable scientific standard, however we have significant reservations, as outlined above.

# Peer review: Reviewer response for version 1

**DOI:** 10.5256/f1000research.24995.r61590

**Published:** 2020-05-26

**Authors:** Elizabeth A. Worthey

**Affiliations:** 1 Center for Computational Genomics and Data Science, Departments of Pediatrics and Pathology, School of Medicine, University of Alabama at Birmingham, Birmingham, AL, USA

This article compares a number of tools released to characterize the presence and nature of repetitive elements from genomic data. The stated goal of the analysis is to generate findings that would help other researchers choose suitable tools and parameters when performing this type of analysis in their own lab. Aspects considered included the ability to call as well as accurately genotype repetitive elements. The analysis is not limited to long disease associated repeats, which has often been the case for obvious reasons.

 The authors compared four different short tandem repeats genotyping tools making use of whole exome sequencing data. They sought to determine genotyping performance and limits. They also examined parameters settings to determine if and how to refine the analysis to increase accuracy. The authors made use of a readily accessible dataset for these analyses.

 As would be expected, they found that all tools showed reasonable performance when genotyping repeats of 3-6 bp in length. As would be expected, they found that accurate homopolymer genotyping was challenging for all tools, with a high error rate being seen cross the board. They found issues nin mono and dinucleotide repeats as would also be expected/has been shown previously. The authors categorized tools based on a number of factors. For example they found that LobSTR made the most calls (which may or may not be a good thing) and was the fastest tool, while RepeatSeq and HipSTR exhibited the lowest heterozygous error rate at low coverage.

 The methods, tools reviewed, and dataset used for testing were clearly presented. The reasoning for selecting those tools was also well laid out. The article cited the current literature in the field.

 The study design was appropriate for the purpose of comparing tools to define specific strengths and weaknesses. It might have been nice to have more of an in depth discussion as to how the various tools differed in terms of their algorithm and how this related to the results and conclusions made. The authors stated that a goal of the analysis would be to generate a framework useful for others looking to compare and contrast tools to support selection for research. As such it might have been nice to include some specifics on ease of installation, levels of support etc. for the various tools etc. Maybe they were all very easy and well supported in that regard.

 The authors provide specific details of the parameters used to run each tool. The majority appear to be run using defaults; there does not seem to have been a huge amount of parameter refinement . This may be due to limitations in what is possible with these tools. The authors provided links to the datasets used for the comparison. The source data used is not provided per se because it is access controlled. The data is, however, a well known publicly available dataset and it should not be very difficult for others to obtain access. People wishing to recapitulate the study would simple have to apply. The authors provided links to the code they used. All combined; these aspects should make for ease of replication by those interested.

 Very minimal statistical analysis and its interpretation was required because of the nature of the analysis presented, but zenodo links to scripts were provided.

 The conclusions drawn were adequately supported by the findings that they presented.

 Testing of the X chromosome for accuracy in variant calling is not a novel approach. Study of the X to explore the genotype and location of repetitive sequences is also not a novel approach. Combining the two approaches to use the X chromosome to specifically examine repetitive sequences has not been published.

 The study is essentially a comparison of tools. As such, it is well organized and executed and would be of interest for people looking to set up pipelines in their lab. As the authors acknowledge most findings gleaned from their analysis were not surprising; they primarily confirmed known constraints or issues with the tools and their analysis confirmed existing knowledge regarding the nature of the repetitive sequences studied. Selection of the X chromosome was a nice way to undertake analysis that would be likely to be adopted by others. Given this background, this work would seem to have value primarily for education purposes to help researchers select and set up tools. It would be useful for that purpose. It does not present much, if anything, in the way of novel generalizable research knowledge on the repetitive nature of the human genome.

Is the work clearly and accurately presented and does it cite the current literature?

Yes

If applicable, is the statistical analysis and its interpretation appropriate?

Yes

Are all the source data underlying the results available to ensure full reproducibility?

Yes

Is the study design appropriate and is the work technically sound?

Yes

Are the conclusions drawn adequately supported by the results?

Yes

Are sufficient details of methods and analysis provided to allow replication by others?

Yes

Reviewer Expertise:

Computational biology. Genetics.

I confirm that I have read this submission and believe that I have an appropriate level of expertise to confirm that it is of an acceptable scientific standard.
